# Significance of Maternal and Cord Blood Nucleated Red Blood Cell Count in Pregnancies Complicated by Preeclampsia

**DOI:** 10.1155/2014/496416

**Published:** 2014-03-06

**Authors:** Shripad Hebbar, Mehak Misha, Lavanya Rai

**Affiliations:** Department of Obstetrics and Gynaecology, Kasturba Medical College, Manipal University, Manipal 576 104, India

## Abstract

*Objectives.* To evaluate the effect of preeclampsia on the cord blood and maternal NRBC count and to correlate NRBC count and neonatal outcome in preeclampsia and control groups. *Study Design.* This is a prospective case control observational study. *Patients and Methods.* Maternal and cord blood NRBC counts were studied in 50 preeclamptic women and 50 healthy pregnant women. Using automated cell counter total leucocyte count was obtained and peripheral smear was prepared to obtain NRBC count. Corrected WBC count and NRBC count/100 leucocytes in maternal venous blood and in cord blood were compared between the 2 groups. *Results.* No significant differences were found in corrected WBC count in maternal and cord blood in cases and controls. Significant differences were found in mean cord blood NRBC count in preeclampsia and control groups (40.0 ± 85.1 and 5.9 ± 6.3, *P* = 0.006). The mean maternal NRBC count in two groups was 2.4 ± 9.0 and 0.8 ± 1.5, respectively (*P* = 0.214). Cord blood NRBC count cut off value ≤13 could rule out adverse neonatal outcome with a sensitivity of 63% and specificity of 89%. *Conclusion.* Cord blood NRBC are significantly raised in preeclampsia. Neonates with elevated cord blood NRBC counts are more likely to have IUGR, low birth weight, neonatal ICU admission, respiratory distress syndrome, and assisted ventilation. Below the count of 13/100 leucocytes, adverse neonatal outcome is quite less likely.

## 1. Introduction

Of late, preeclampsia, a leading cause for pregnancy related morbidity and mortality, has been classified as early onset (which develops before 34 weeks of gestation) and late onset (which develops after 34 weeks). Early onset preeclampsia is considered as a fetal disorder that is typically associated with reduction in placental volume, intrauterine growth restriction, abnormal uterine and umbilical artery Doppler, low birth weight, multiorgan dysfunction, perinatal death, and adverse maternal and neonatal outcomes. Late onset preeclampsia is considered a maternal condition; a result of an underlying maternal constitutional disorder, and is more often associated with normal placenta, larger placental volume, along with a normal fetal growth [1]. Preeclampsia when associated with placental hypoperfusion results in hypoxic response in developing fetus in the form of increased erythropoiesis and release of immature erythrocytes [2]. Several studies have shown an increased number of nucleated red blood cell (NRBC) counts in the cord blood of newborns of preeclamptic mothers [3, 4].

Nucleated red blood cells were first noted in 1871 to be present in the peripheral blood of neonates [5]. Until the sixth and seventh weeks of gestation, all fetal red blood cells are nucleated. By the twelfth week of gestation, nucleated red blood cell counts decline [6]. Although rarely found circulating in older children [7], they are commonly seen in the blood of newborns. The rise in number of circulating nucleated red blood cells can be the result of any stimulus which increases erythropoietic activity or a sudden release from the marrow storage pools. Any hypoxic event which induces a fetal compensatory response in the form of exaggerated erythropoiesis primarily in the bone marrow results in influx of immature red blood cells into fetal circulation. Nucleated red blood cells are a potentially useful tool in estimating the degree of intrauterine hypoxia. Elevated neonatal red blood cell counts have been shown in hypoxic conditions such as erythroblastosis fetalis, maternal diabetes mellitus, acute fetal distress, intrauterine growth restriction, premature rupture of membranes, and chorioamnionitis. Fetal NRBCs are also found circulating in maternal blood during pregnancy.

This study was undertaken to assess the extent to which NRBCs were associated with adverse neonatal outcome in preeclampsia and healthy pregnant women and see if this count is a reliable marker to predict early neonatal outcome. Another aspect of the study was determination of the difference between the cord blood NRBC counts of preeclamptic and healthy pregnant patients. Maternal NRBC count was studied as in some studies it was postulated that disturbed fetomaternal cell trafficking in preeclampsia leads to raised maternal NRBCs.

## 2. Methods

This was a prospective case control observational study done in the Department of Obstetrics and Gynaecology at Kasturba Medical Hospital, which is a teaching hospital for undergraduate and postgraduate students studying at Manipal University, India. Institutional ethical committee clearance was obtained at the start of the study. Singleton pregnancies with preeclampsia with gestational period ≥28 weeks were included as cases and low risk healthy pregnancies as controls for comparison.

### 2.1. Sample Size Calculation

We wanted to know whether nucleated RBC counts in cord blood of preeclamptic mothers significantly differ from NRBC counts of healthy pregnant women. We referred to the published [3] mean and standard deviation for nucleated RBC count per 100 WBC in umbilical blood of normal healthy neonates (8.6 ± 7.01). Saracoglu et al. [8] reported that 14 NRBCs/100 leucocytes in the cord blood demarcate hypoxic babies from healthy newborns and hence we hypothesized that mean NRBC count of 14 would be significantly different from the norm. With a desired level of power of 90% and level of significance of 0.05, the sample size was calculated using the following formula:

(1) $$\begin{array}{c} n={\left(\frac{\left(z_{\alpha }+z_{\beta }\right)\sigma }{\mu_{1}-\mu_{0}}\right)}^{2}, \end{array}$$

where *z*_*α*_ = 1.96 (critical value that divides the central 95% of *z* distribution from 5% in the tails), *z*_*β*_ = 1.28 (critical value that separates the lower 10% of distribution from upper 90%), *σ* = 7.01 (standard deviation), and *μ*_1_ − *μ*_2_ = 14 − 8.6 = 5.4 (difference of two means).

The minimum sample size required accordingly was 18 subjects in case and control groups; however, we decided to recruit 50 subjects for each group.

**Figure 1:**
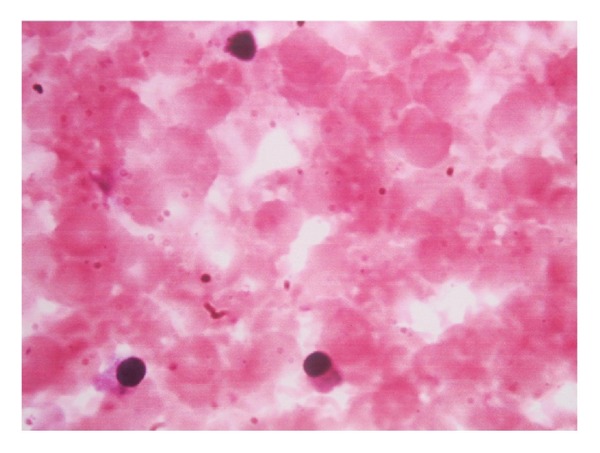
Photomicrograph of nucleated red blood cells in cord blood of a newborn.

Preeclampsia was diagnosed when the blood pressure was ≥140/90 mm Hg and there was associated proteinuria of at least 30 mg/L (1+ on dipstick) in two random urine samples or 300 mg in 24 hours at ≥20 weeks of gestation. In the absence of proteinuria, preeclampsia was diagnosed when blood pressure was ≥140/90 mm Hg in association with persistent cerebral symptoms, epigastric or right upper quadrant pain plus nausea or vomiting, fetal growth restriction or with thrombocytopenia, and elevated liver enzymes. Cases with fetal distress (taken as presence of late decelerations of the fetal heart, decreased fetal heart rate variability, fetal bradycardia, or persistent fetal tachycardia), prolonged rupture of membranes (more than 6 hours), multifetal gestation, diabetes mellitus, anaemia, Rh isoimmunisation, and stillbirth were excluded as these conditions themselves may produce acute rise in fetal NRBC count. Immediately after delivery, 1 mL of umbilical cord blood was collected in a tube containing 1.5 mg ethylenediaminetetraacetic acid (EDTA). 1 mL of maternal venous blood was collected in the same manner. Using automated cell counter, total leucocyte count was obtained. For the purpose of making peripheral smear, a drop of blood sample was placed towards one end of a glass slide and spreader glass slide was placed at 45 degree inclination and in one uniform motion blood was smeared on the rest of the slide. Slide was allowed to dry and was then covered with Leishman's stain. After 5 minutes, stain was diluted with distilled water and was mixed on slide. Slide was allowed to absorb stain for 15 minutes and was then washed in gentle stream of water. Under pathologist's supervision, smear was focused under high power microscope and RBCs (nucleated) were counted against 100 WBCs (Figure 1).

Corrected WBC count was obtained by using the following formula:

(2) Corrected  WBC  count   =  (Uncorrected  WBC  count×100)(NRBC  per  100  leucocytes+100).

We studied various neonatal outcome measures such as APGAR scores, birth weights, need for assisted ventilation, NICU admission, and respiratory distress syndrome (RDS). We defined low birth weight (LBW) as weight less than 2.5 kg at birth and intrauterine growth restriction (IUGR) as birth weight less than tenth percentile for corresponding gestational age. Criteria for diagnosis of RDS in the newborn included any of the following: (i) presence of rapid, noisy, or difficult breathing; (ii) respiratory rate >60/min; (iii) cyanosis, chest retraction, or grunting; (iv) need for mechanical ventilation for more than 24 hours; (v) diffuse alveolar damage as shown by chest radiography; (vi) need for surfactant therapy.

Statistical analysis was done using Chi-square test to compare the categorical variables and independent Student's *t*-test for comparing continuous variables with Gaussian distribution, and Mann-Whitney test was used to compare continuous variables with non-Gaussian distribution. Receiver operating curves were used to define cutoffs of NRBC count/100 leukocytes for each neonatal variable.

## 3. Results

A total of 100 women were studied out of which 50 preeclamptic women were cases and 50 healthy pregnant women were controls.  Table 1 shows maternal and neonatal characteristics of both groups.

The difference in mean maternal ages in preeclampsia and control groups was not statistically significant. The mean gestational age at the time of delivery in preeclamptic patients was significantly lower compared to normal controls. There was no significant difference between two groups regarding the mode of delivery. The number of primiparae and multiparae were comparable in both study and control groups. Both maternal and cord blood corrected WBC counts were matching in study and control groups. Neonatal characteristics revealed significant differences between occurrence of low birth weight babies (less than 2500 grams), intrauterine growth restriction (IUGR), low APGAR scores, need for assisted ventilation, and respiratory distress syndrome (RDS). There was also increased need for admission to neonatal intensive care unit (NICU) for the babies born to preeclamptic mothers.

We also studied the influence of gestational age on the cord blood NRBC count by regression analysis. The relation between these two variables was indicated by the following equation:

(3) $$\begin{array}{c} \frac{\text{Cord}\;\text{blood}\;\text{NRBC}}{100\;\text{WBC}\;}=292.56-7.389*\text{Gestational}\;\text{Age}. \end{array}$$

**Figure 2:**
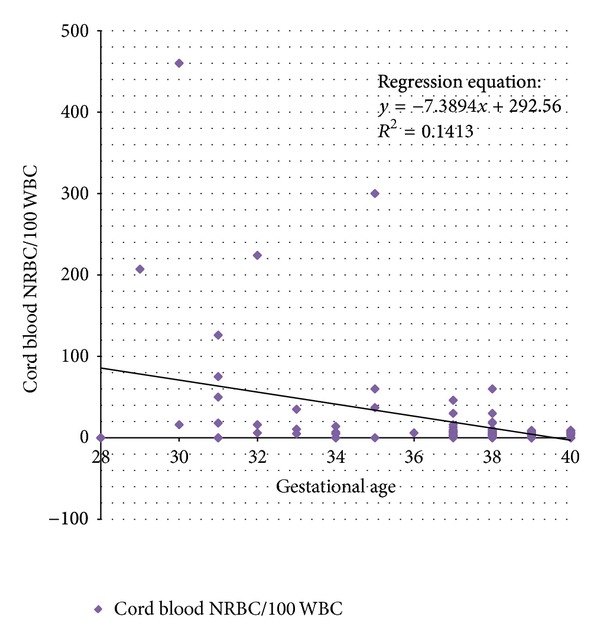
Effect of advancing gestational age on cord blood NRBC count.

The goodness of fit in linear regression analysis is explained by coefficient of regression (*R* ^2^) which varies between 0 and 1. Values close to 1 indicate good correlation between two variables and those close to zero indicate poor correlation. The coefficient of regression in our study was 0.1413, indicating that only 14% of variation in NRBC count was explained by gestational age (Figure 2).

It was found that there was no significant difference in the cord blood nucleated red blood cells of the patients who had vaginal or caesarean delivery (mean cord blood NRBCs count (mean ± SD): vaginal delivery (12.8 ± 32.7), caesarean delivery (30.08 ± 76.1), *P* value: 0.20—statistically not significant).

Significantly higher counts of NRBC were found in the cord blood of preeclamptic women than in controls (Table 2). Though variation is noted in the mean cord blood nucleated red blood cell count/100 leucocytes in both preeclampsia and control groups, the mean difference between the two groups was found to be statistically significant (*P* = 0.006), but not in maternal blood per se (*P* = 0.214).

There were 26 women in preeclampsia group who delivered at term and their cord blood NRBC counts were compared with control group to eliminate the confounding effect of preterm gestation on elevated NRBC count. The difference of the counts between the term preeclampsia patients and the control group was still significant (12.8 ± 14.3 versus 5.9 ± 6.3, *P* = 0.02). Significant difference existed between cord blood nucleated red blood cell counts between neonates who had IUGR, those admitted to NICU, those who needed assisted ventilation, or those who had RDS and those who did not have these within the preeclampsia group (Table 3). This means that the cord blood NRBCs are elevated more in babies manifesting hypoxia consequences. Out of the 33 non-IUGR neonates of preeclampsia mothers, 2 (6%) had low APGAR, 4 (12%) had RDS, 4 (12%) needed assisted ventilation, and 8 were admitted to NICU in contrast to 17 IUGR neonates of preeclampsia mothers, 10 (58%) of whom had RDS, 5 (29.4%) had low APGAR, 6 (35.3%) needed assisted ventilation, and 13 (76.5%) needed NICU admission.

**Figure 3:**
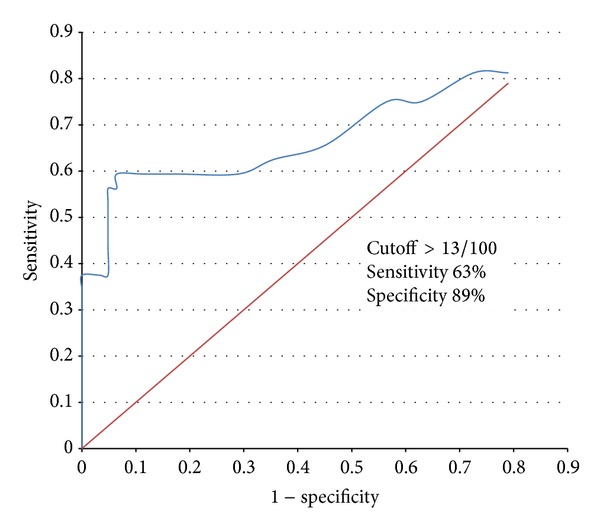
ROC analysis of overall adverse neonatal outcome according to NRBC cutoff of >13/100.

Using the ROC curve, cord blood NRBC count cutoff was calculated for overall neonatal variables studied (Figure 3). The graph was generated by comparing sensitivity (*y*-axis) against false positive rate (i.e., 1 − specificity) in *x*-axis for various cut-off points of cord blood NRBC levels. The validity of a ROC plot depends upon the area under the curve (AUC) which in our study was 0.71 indicating fair association between test statistics and outcome status. One or more adverse neonatal outcomes could be predicted with cord blood NRBC count >13/100 leucocytes with a sensitivity of 63% and a specificity of 89%.  Table 4 shows various neonatal outcomes using cord blood NRBC cutoff of 13. It can be seen that cord blood NRBC count ≤13 is less likely to be associated with adverse neonatal outcomes.

## 4. Discussion

It has been stated that the inability of cytotrophoblasts to differentiate correctly and subsequent failure to invade the uterus and its arterioles efficiently in preeclampsia lead to a relatively hypoxic placenta [9]. So compensatory mechanisms like enhanced production of erythrocytes (nucleated RBCs) are activated to counteract this imbalance [10].

Bayarm and coworkers from Departments of Pediatrics, Uludağ University, Bursa, Turkey, conducted a study on NRBCs in 43 preeclamptic and 25 healthy pregnant women [11]. Preeclamptic women were further subgrouped based on the presence or absence of intrauterine growth restriction. The NRBC count in these subgroups differed significantly and without IUGR. A nucleated red blood cell count of 18.5 or above could predict fetal asphyxia with a sensitivity of 94.4% and a specificity of 80%. Similar observations were made in the present study, though the ROC analysis gave a lower cutoff for NRBC.

A raised NRBC count in newborn also serves as an indicator of perinatal asphyxia of both acute and chronic origins. In a Turkish study [8], mean cord blood NRBC count/100 leucocytes was found to be 11.18 ± 4.92 for acute fetal distress group (*n* = 11) and 24.43 ± 20.05 for chronic fetal distress group (*n* = 11). Both values were significantly higher than the control group (*P* < 0.05; *P* < 0.005, resp.). They also determined that a cutoff of 14 NRBCs/100 leucocytes in the cord blood discriminated the acidotic fetuses from the nonacidotic ones with sensitivity of 87% and specificity of 81%.

Salafia et al. from Department of Pathology, Montefiore Medical Center, Bronx, New York, reported that the NRBC count was independent of gestational age in well-grown, nonacidotic, nondepressed preterm infants [12]. In the present study too, the cord blood nucleated RBC count did not vary with the gestational age in the preeclampsia group or the control group or the two groups taken together. In the same study, the number of NRBCs was found to be correlated with umbilical cord blood pH. They concluded that the number of cord blood nucleated red blood cells could be a useful marker in determining both the duration and degree of asphyxia injury. Ghosh and coworkers from All India Institute of Medial Sciences, New Delhi, India, [3] demonstrated a statistically significant correlation between NRBC count/100 leucocytes in the cord blood and presence of IUGR, umbilical artery pH at birth, and APGAR score at 1 minute in their study comprising 75 subjects. They suggested that NRBC count/100 leucocytes in the cord blood can be used as a reliable index of early neonatal outcome.

Maternal nucleated red blood cells have been studied in preeclampsia patients and have been shown to be raised compared to counts in maternal blood in controls suggesting disturbed fetomaternal cell trafficking in preeclampsia. This may help us understand the pathophysiology of this disease better. However, in the present study, no significant difference was found between nucleated red blood cell count of preeclamptic mothers and that of healthy pregnant women. Studies incorporating advanced methods (such as FISH, magnetic activating cell sorting protocol, etc.) have demonstrated significant increase in the traffic of nucleated fetal cells in the preeclamptic subjects compared to the controls [13, 14].

A low first minute APGAR score in newborn is also associated with high nucleated RBC level [15]. It has been observed that caesarean delivery for fetal distress, IUGR, oligoamnios, low APGAR scores, and fetal academia (as indicated umbilical arterial pH < 7) were associated with statistically significant increases in nucleated red blood cell counts [16, 17]. Neonates with higher NRBC counts were more likely to be admitted to the NICU according to the results of the present study. It also has been observed that NRBC levels tend to remain elevated for longer time during neonatal period among neonates with chronic fetal asphyxia compared to those babies who had acute fetal distress during delivery [18]. In the present study, cord blood NRBC count was significantly raised in cases of IUGR babies compared to appropriate for gestational age (AGA) babies within the cases of preeclampsia. Thus, NRBC count may help to distinguish growth restricted babies from constitutionally small neonates.

In the present study, it was found that adverse neonatal outcome can be expected when NRBC count/100 leucocytes in the cord blood of the neonate exceeds 13 with a sensitivity of 63% and a specificity of 89%. According to the results of the present study, it may be suggested that absence of elevated NRBCs may help in ruling out adverse neonatal outcome. At higher cut-off levels, the sensitivity increases and specificity decreases.

## 5. Conclusion

Cord blood nucleated red blood cells are significantly raised in preeclampsia and are associated with adverse early neonatal outcome. Neonates with elevated cord blood NRBC counts are more likely to have IUGR, low birth weight, neonatal ICU admission, respiratory distress syndrome, and assisted ventilation. Below the count of 13/100 leucocytes, adverse neonatal outcomes are quite less likely.

## 6. Limitations of the Study

1. Repeat neonatal NRBC counts could have been done during the NICU stay. If repeated at regular intervals after birth, their persistence and trend could be studied in the preeclampsia cases and comparison could be made between neonates with adverse outcomes and healthy infants.
2. Umbilical venous blood gas analysis and fetal scalp pH were not done. Relation of cord blood NRBCs with fetal acidosis could not be studied.
3. Neonates with acute fetal distress during labour were excluded to eliminate bias as this event is associated with rise in NRBC count. However, it has been opined that such a rise is relatively less compared to those babies with chronic asphyxia, and if this subgroup of babies was included in the study, this phenomenon could be documented. This is of importance because cerebral palsy due to acute birth asphyxia is of medicolegal importance.

## Figures and Tables

**Table 1:** Comparative study of maternal and neonatal characteristics in preeclampsia and control groups.

Characteristics	Preeclampsia (*n* = 50)	Controls (*n* = 50)	*P* value
(A) Maternal profile			
Age in years (mean ± SD)	26.8 ± 4.5	27.3 ± 3.8	0.537*
Systolic blood pressure—mm of Hg (mean ± SD)	155.0 ± 9.1	115.9 ± 12.7	0.00*
Diastolic blood pressure—mm of Hg (mean ± SD)	93.7 ± 6.1	72.5 ± 8.5	0.00*
Corrected WBC count/mm^3^ (mean ± SD)	11,755 ± 4,837	11,709 ± 5,965	0.519*
Gestational age at delivery in weeks (mean ± SD)	35.2 ± 3.1	38.5 ± 1.1	0.00*
Caesarean section (*n*/%)	34 (68)	25 (50)	0.103**
Vaginal delivery (*n*/%)	16 (32)	25 (50)	
Nullipara (*n*/%)	27 (54)	18 (36)	0.107**
Multipara (*n*/%)	23 (46)	32 (64)	
(B) Neonatal profile			
Corrected cord blood WBC count/mm^3^ (mean ± SD)	9,875 ± 4,920	11,157 ± 4,406	0.171*
Birth weight in grams (mean ± SD)	2171.0 ± 805.3	2976.1 ± 473.3	0.00*
Birth weight <2500 g (*n*/%)	29 (58)	1 (2)	0.00**
IUGR (*n*/%)	17 (34)	0 (0)	0.00**
APGAR at 5 min <7 (*n*/%)	8 (16)	1 (2)	0.031**
Assisted ventilation (*n*/%)	10 (20)	1 (2)	0.008**
RDS (*n*/%)	14 (28)	0 (0)	0.00**
NICU admissions (*n*/%)	21 (42)	1 (2)	0.00**
Neonatal deaths (*n*/%)	1 (2)	0 (0)	1.00**

*Independent *t*-test; **Chi-square test.

**Table 2:** Comparison of NRBC counts/100 leucocytes in preeclampsia and control groups.

	Preeclampsia group (*n* = 50)	Control group (*n* = 50)	*P* value*
Maternal NRBC count (mean ± Std deviation)	2.4 ± 9.0	0.8 ± 1.5	0.214
Cord blood NRBC count (mean ± Std deviation)	40.0 ± 85.1	5.9 ± 6.3	0.006

*Mann-Whitney *U* test.

**Table 3:** Association of increased cord blood NRBC count with different neonatal outcomes studied within cases of preeclampsia group (*n* = 50).

Neonatal outcome	*N*	Cord blood NRBC/100 WBC (mean ± SD)	*P* value*
IUGR			
Present	17	83.0 ± 133.4	0.004
Absent	33	17.9 ± 26.8	
APGAR			
Good	8	50.0 ± 68.1	0.203
Low	42	38.2 ± 88.6	
Need for assisted ventilation			
Present	10	90.6 ± 142.7	0.008
Absent	40	27.5 ± 59.9	
NICU admission			
Present	21	77.6 ± 121.6	0.027
Absent	29	12.9 ± 17.0	
Respiratory distress			
Present	14	109.6 ± 139.4	0.002
Absent	36	13.0 ± 15.6	
Neonatal death			
Present	1	207.0	#
Absent	49	36.7 ± 82.5	
One or more adverse outcomes			
Present	26	67.3 ± 111.5	0.014
Absent	24	10.6 ± 13.1	

#: number too small to apply statistical tests.

*P value calculated by Mann-Whitney test.

**Table 4:** Distribution of different neonatal outcomes (in all patients studied, *n* = 100) with cord blood NRBC/100 WBC cutoff of 13 (>13 and ≤13, resp., in each neonatal outcome).

Parameter	Cord blood NRBC >13/100 WBC	Cord blood NRBC ≤13/100 WBC	*P* value*
	*n* (%)	*n* (%)	
IUGR			
Present	11 (44%)	6 (8%)	0.000
Absent	14 (56%)	69 (92%)	
APGAR			
Low	6 (24%)	3 (4%)	0.007
Good	19 (76%)	72 (96%)	
Need for assisted ventilation			
Needed	8 (32%)	3 (4%)	0.001
Not needed	17 (68%)	72 (96%)	
NICU admission			
Needed	14 (56%)	8 (10.7%)	0.000
Not needed	11 (44%)	67 (89.3%)	
Birth weight (g)			
<2500	18 (72%)	12 (16%)	0.000
≥2500	7 (28%)	63 (84%)	
Respiratory distress			
Present	10 (40%)	4 (5.3%)	0.000
Absent	15 (60%)	71 (94.7%)	
Neonatal death			
Present	1 (4%)	0 (0%)	0.25
Absent	24 (96%)	75 (100%)	
One or more adverse outcomes			
Present	17 (68%)	10 (13.3%)	0.000
Absent	8 (32%)	65 (86.7%)	

*Chi-square test.
